# Diagnosis of *Streptococcus pneumoniae* infection using circulating antibody secreting cells

**DOI:** 10.1371/journal.pone.0259644

**Published:** 2021-11-12

**Authors:** Shuya Kyu, Richard P. Ramonell, Merin Kuruvilla, Colleen S. Kraft, Yun F. Wang, Ann R. Falsey, Edward E. Walsh, John L. Daiss, Simon Paulos, Gowrisankar Rajam, Hao Wu, Srinivasan Velusamy, F. Eun-Hyung Lee

**Affiliations:** 1 Division of Pulmonary, Allergy, Critical Care and Sleep Medicine, Department of Medicine, Emory University School of Medicine, Atlanta, Georgia, United States of America; 2 Division of Infectious Diseases, Department of Medicine, Emory University, Atlanta, Georgia, United States of America; 3 Department of Pathology and Laboratory Medicine, Emory University School of Medicine, Atlanta, Georgia, United States of America; 4 Division of Infectious Diseases, Department of Medicine, University of Rochester Medical Center, Rochester, New York, United States of America; 5 Rochester General Hospital, Rochester, New York, United States of America; 6 Center for Musculoskeletal Research and Department of Orthopaedics, University of Rochester Medical Center, Rochester, New York, United States of America; 7 MicroB-plex, Inc., Atlanta, Georgia, United States of America; 8 Merck & Co., Inc., Kenilworth, New Jersey, United States of America; 9 Department of Biostatistics and Bioinformatics, Emory University, Atlanta, Georgia, United States of America; 10 Division of Bacterial Diseases, National Center for Infectious Diseases, Centers for Disease Control and Prevention, Atlanta, Georgia, United States of America; Instituto Butantan, BRAZIL

## Abstract

**Background:**

*Streptococcus pneumoniae* infections cause morbidity and mortality worldwide. A rapid, simple diagnostic method could reduce the time needed to introduce definitive therapy potentially improving patient outcomes.

**Methods:**

We introduce two new methods for diagnosing *S*. *pneumoniae* infections by measuring the presence of newly activated, pathogen-specific, circulating Antibody Secreting Cells (ASC). First, ASC were detected by ELISpot assays that measure cells secreting antibodies specific for signature antigens. Second, the antibodies secreted by isolated ASC were collected *in vitro* in a novel matrix, MENSA (media enriched with newly synthesized antibodies) and antibodies against *S*. *pneumoniae* antigens were measured using Luminex immunoassays. Each assay was evaluated using blood from *S*. *pneumoniae* and non-*S*. *pneumoniae*-infected adult patients.

**Results:**

We enrolled 23 patients with culture-confirmed *S*. *pneumoniae* infections and 24 controls consisting of 12 non-*S*. *pneumoniae* infections, 10 healthy donors and two colonized with *S*. *pneumoniae*. By ELISpot assays, twenty-one of 23 infected patients were positive, and all 24 controls were negative. Using MENSA samples, four of five *S*. *pneumoniae*-infected patients were positive by Luminex immunoassays while all five non-*S*. *pneumoniae*-infected patients were negative.

**Conclusion:**

Specific antibodies produced by activated ASC may provide a simple diagnostic for ongoing *S*. *pneumoniae* infections. This method has the potential to diagnose acute bacterial infections.

## Introduction

*Streptococcus pneumoniae (S*. *pneumoniae)* is the most frequent cause of community-acquired pneumonia (CAP) [1], and associated with bacteremia in 25% of cases [2]. Globally, infection with *S*. *pneumoniae* is responsible for 1.6 million deaths annually [3], and is a major cause of vaccine-preventable deaths among children [4]. Furthermore, *S*. *pneumoniae* is also a leading cause of morbidity and mortality among the elderly [5].

The early identification of *S*. *pneumoniae* infections can affect outcomes for individual patients and the population at large. Diagnosis is essential for antibiotic de-escalation in infected patients since overuse of broad-spectrum antibiotics contributes to antimicrobial resistance [6]. However, conventional culture-based identification and molecular assays have recognized limitations. The current gold standard for diagnosing *S*. *pneumoniae* in CAP infections consists of positive sputum cultures, blood cultures, or urinary antigen tests in adults with pneumonia [7]. Culture-based diagnosis requires bile solubility and optochin susceptibility testing, but the reliability can on rare occasions be compromised by bile-insoluble [8, 9] and optochin-resistant strains. In addition, prior antimicrobial treatment may result in false negative diagnoses [4]. Since *S*. *pneumoniae* is a known colonizer of the respiratory tract, direct pathogen detection in sputum alone does not define it as the causative pathogen [10]. Initially, urinary antigen testing were thought to have high sensitivity and specificity; however, multiple later studies showed sensitivity of 60-65% and much lower after the introduction of the 13-valent polysaccharide vaccine (PCV13) [11]. Additionally, serological assays for *S*. *pneumoniae* have been problematic since they rely on both acute and convalescent titers of antibody responses against capsular polysaccharides. Moreover, exposure to *S*. *pneumoniae* early in life induces seroconversion against homologous capsular polysaccharides thereby limiting the diagnostic utility of serum-borne antibodies [12]. Due to the lack of sensitive and specific diagnostic assays, guidelines for CAP have discouraged use of serological diagnosis in non-hospitalized patients [13].

An alternative approach is to capture antibodies secreted by newly activated antibody secreting cells (ASC) that emerge into the blood during the acute infection. These circulating ASC ultimately migrate to the bone marrow, spleen, or lung to clear the infection and provide long-term protective antibodies [14]. Interestingly, only during active infection do the ASC circulate in the blood. Thus, measuring antibodies from circulating ASC is an underappreciated approach for microbiologic diagnosis [15–17]. The transient appearance of the ASC in blood occurs in both bacterial and viral infections [16–18]. ASC appear as early as two days post-symptom onset (DPSO) and disappear when the infection has resolved [17]. These attributes have sparked interest in the development of assays using pathogen-specific ASC for diagnosis of active infections [18–20]. Direct enumeration of ASC by ELISpot assays or measurement of antibodies from circulating ASC collected *in vitro* in a novel matrix, MENSA (media enriched with newly synthesized antibodies), have been shown to be effective diagnostics [16–20].

In this paper, we evaluated the circulating ASC response to four signature antigens in adult patients with *S*. *pneumoniae* infection by both ELISpot and MENSA assays. We show that ASC circulating during the acute infection secrete anti-pneumococcal antibodies, and that this approach could be potentially utilized to identify the often difficult to diagnose *S*. *pneumoniae* infections.

## Methods

### Institutional approval

All research was approved by the Emory Institutional Review Board. Informed consent was obtained from all participants.

### Human subjects

Patients were enrolled from Emory University Hospital, Emory University Hospital Midtown, Emory Saint Joseph’s Hospital, and Grady Memorial Hospital in Atlanta, Georgia. We enrolled patients 21-85 years old who had cultures positive for *S*. *pneumoniae* (n = 23) or other pathogens (n = 12). Patients on immunosuppressive medications were excluded; however, HIV patients on chronic anti-retroviral therapy were included. Ninety-one healthy adults were enrolled as controls and evaluated for colonization. All protocols were approved by the Emory institutional review board. Written consent was obtained from each patient or a legally authorized representative (LAR), oftentimes a family member, if the patient was not able to consent.

### Blood culture diagnosis and patient recruitment

Blood was collected from patients with positive cultures for *S*. *pneumoniae* and non-*S*. *pneumoniae* pathogens. Bacterial cultures were performed by the Emory University Hospital microbiology laboratory. Species were identified using standard biochemical and molecular methods described previously [21]. Briefly, peripheral blood samples were collected in bottles containing culture medium and were continuously monitored during incubation at 37°C for five days. For respiratory cultures and wounds, samples were plated upon receipt and grown in CO_2_ incubators. Once growth of bacterial colonies was detected, Gram-staining and subcultures were performed to enable genus and species identification. Patients with positive cultures were enrolled between 1-17 days after confirmation. Six patients who only produced samples after 4 days post-confirmation were used to determine the window of collection in Fig 3. Patient information for those with *S*. *pneumoniae* and non-*S*. *pneumoniae* infections is presented in Table 1.

**Table 1:** Demographic and clinical information for infected patients.

Characteristic	*S. pneumoniae* Positive (n = 23)	*S. pneumoniae* Negative (n = 12)
**Age: years (range)**	53±17 (27-83)	48±13 (24-70)
**Gender: number (%)**		
Male	15 (68%)	5 (41%)
**Symptoms – no. (%)**		
Fever	14 (65%)	7 (58%)
Cough	14 (64%)	6 (50%)
Sputum	13 (59%)	4 (33%)
Fatigue	15 (70%)	7 (58%)
Malaise	19 (90%)	10 (91%)
ICU	7 (35%)	6 (50%)
Ventilator	2 (10%)	1 (8%)
Sepsis	4 (25%)	5 (42%)
**Comorbidities – no. (%)**		
Transplant	1 (5%)	3 (27%)
Cancer	6 (30%)	2 (18%)
HIV	4 (24%)	2 (18%)
HBV	0 (0%)	0 (0%)
Splenectomy	1 (5%)	0 (0%)
B cell biologics	0 (0%)	0 (0%)

### Nasopharyngeal swabs and *lytA* PCR

Nasopharyngeal swab samples were obtained from 91 healthy adults and PCR targeting the major autolysin gene, *lytA*, was performed as described previously [22].

### Peripheral Blood Mononuclear Cell (PBMC) isolation

Peripheral blood (20 mL) was collected in sodium heparin tubes and processed within 8 hours. PBMC were isolated by density gradient centrifugation at 800×g for 20 minutes. The PBMC were washed 5 times with RPMI at 500×g for five minutes. Viability was assessed by trypan blue exclusion; live cells were counted using a Bio-Rad TC20^TM^ automated cell counter. Cells were incubated in RPMI with 10% fetal bovine serum (R10) at 37°C with 5% CO_2_ until use in the ELISpot assay.

### MENSA generation

PBMC were resuspended in R10 at 2.5×10^6^ cells/mL; 5×10^5^ PBMC (200 μL) were added to culture wells coated with bovine serum albumin (BSA) and incubated for 16-24 hours, 37°C, 5% CO_2_. MENSA supernatant (150 μL) was collected from each well and stored frozen at -80°C until use.

### Antigens

PsaA (Serotype 6B) was generously provided by Edwin Ades (Sanofi-Pasteur, Toronto, Ontario, Canada [23]). PspA (clades 2 (Rx1), 3 and 4 (JCP#56)) were obtained from BEI Resources (NR-33178, NR-33179, and NR-33180) and CWPS was purchased from Statens Serum Institut (1:1 mixture of a noncapsulated former serotype 2 strain and 22F-R-LSA/ICS) (Cat. #68866). Recombinant PcsB (serotype 4, TIGR4) was synthesized and purified by GenScript as described previously [24, 25].

### Enzyme-Linked Immunospot (ELISpot) assay

ELISpot plates (Millipore) were coated with antigens diluted in phosphate-buffered saline (PBS): PcsB (50 μg/mL), PsaA (50 μg/mL), CWPS (50 μg/mL), and control wells with 2% BSA and performed similarly as previously described [26]. For PspA, equal amounts of clade 2, 3, and 4 proteins were combined in a single well (final concentration 50 μg/mL). Total immunoglobulin wells were coated with polyclonal anti-human IgM, IgG, or IgA, each at 10 μg/mL, in separate wells (Life Technologies). The plates were incubated 2 hours at 37°C, or overnight at 4°C in a humidified chamber. Coated plates were washed 3 times with 200 μL R10, and blocked with R10, 2 hours, 37°C. PBMC (250,000-500,000) were incubated on the plates for 16-24 hours. For detection, the plates were washed with PBS-0.1% Tween (PBS-T) 6 times and incubated with alkaline phosphatase-conjugated anti-IgM, anti-IgA, or anti-IgG antibody (1 μg/mL; Jackson Immunoresearch) for 2 hours, RT. The plates were washed 3 times with PBS-T and soaked in PBS-T for 1 hour and then developed using the Vector Blue Alkaline Phosphatase Substrate kit (SK-5300). Spots were visualized and enumerated using a CTL Immunospot reader.

### Exclusion of indeterminate assays

Assays with ≥4 positive spots in the negative control wells were indicative of non-specific antibody binding. Assays with <100 spots per 500,000 PBMC in the total IgG well were considered indeterminate and excluded from the final analysis.

### Luminex immunoassay antigen-bead conjugation

Pneumococcal protein antigens PcsB, PsaA, and PspA were covalently conjugated to carboxylate-modified, spectrally-distinct Luminex microspheres (beads) using the carbodiimide reaction [27]. Beads (12.5×10^6^ beads/mL) were activated in 10 μL/mL of sodium phosphate buffer (pH6.2) (Cat#S0741, Sigma-Aldrich, St. Louis, MO) containing 2.5 mg of N-(3-Dimethylaminopropyl)-N′-ethylcarbodiimide (EDC, Cat#E7750, Sigma-Aldrich) and 2.5 mg of N-Hydroxysuccinimide (NHS, Cat#130672, Sigma-Aldrich). After 20 min incubation, RT, with inversion and rotation, beads were harvested by centrifugation at 12,000×*g* for 5 min and resuspended in 500 μL 2-(N-Morpholino)ethanesulfonic acid hydrate (MES, Cat#M8250, Sigma-Aldrich) containing the protein antigen. After 2h incubation, RT, the beads were washed once with blocking buffer (PBS-1% BSA; Cat#05470, Sigma-Aldrich), resuspended in 500 μL blocking buffer, and incubated for 30 min, RT, with inversion and rotation. Beads were washed twice with blocking buffer and refrigerated in 1 mL storage buffer (PBS-0.5% BSA).

### *S*. *pneumoniae* multi-antigen luminex immunoassay

Each assay plate included a standard reference serum (CDC 887, In-house human polyclonal serum), diluted 4-fold for 8 dilutions starting at 1/20, and an internal quality control (QC) serum. Test serum samples were diluted 2-fold for 7 dilutions starting at 1/100. IgG-free human serum (Sigma-Aldrich), and assay buffer blanks were used as controls. All dilutions of QC samples, reference standards, and samples were carried out in 96-well round bottom titer plates (Dilution plate, Cat#CLS3799, Sigma-Aldrich). 96-well multiscreen HTS filter plates (MABVN1250, Millipore Corp, Billerica, MA), pre-wet with 100 μL/well assay buffer (PBS-0.1% BSA) were aspirated and antigen-conjugated beads (2500 beads/region/well; 25 μL/well) were added. From the dilution plate, 25 μL reference standards, QC samples and serum samples were transferred to the filter plate with beads and incubated in the dark for 30-50 min, RT, with agitation. The plate was aspirated and washed three times with 100 μL assay buffer. To each well, 50 μL of a 1/200 dilution of R-phycoerythrin-conjugated Goat anti-human Fcγ specific IgG (GTIGF-001, Moss Inc., Pasadena, MD) in PBS was added and incubated for 10-30 min, RT, with agitation. After 3 washes, the beads were resuspended in 130 μL assay buffer. The plate was read in a Luminex 200 reader (Luminex Corp., Houston, TX). The reporter signal value was expressed as the median fluorescent intensity (MFI) and increased monotonically with bead-bound antibody density.

### Data analysis

Masterplex CT/QT data-analysis software (MiraiBio) was used for data reduction and analysis. The program utilizes a four-parameter, logistic-log (4-PL), robustly weighted function to model the test curves from the MFI of dilution points of the reference standard. Sample concentrations were calculated by interpolating the MFI to the reference standard curve. Standard curve fitting, sample concentrations and QC criteria were calculated independently for each antigen.

## Results

### Study subjects

Twenty-three patients with *S*. *pneumoniae*-positive infections and twelve subjects with infections caused by other organisms (*S*. *pneumoniae*-negative) were enrolled, along with 91 healthy controls. Of those with *S*. *pneumoniae* infections, sixteen were bacteremic, five were positive by sputum culture only, and two had positive wound cultures. The primary source of *S*. *pneumoniae* infection was pneumonia in nineteen patients, bronchitis in one, sinusitis in one, paraspinal abscess in one, and skin and soft tissue infection in one. Eighteen patients had lobar pneumonia while one had pneumonia with empyema. Most patients initiated antimicrobial therapy at the time of admission. All non-pneumococcal infections were bacteremic, and included five with *Staphylococcus aureus*, one each with *Klebsiella pneumoniae*, *Enterococcus faecium*, *Escherichia coli*, yeast, and three unidentified bacteremias (Gram-variable rods, *Staphylococcus* species, alpha-hemolytic *Streptococcus* species). Among the healthy subjects, two tested positive for *S*. *pneumoniae* nasopharyngeal colonization by *lytA* qPCR; Ct values were 24.55 and 35.14 for COL1 and COL2, respectively. Peripheral blood was collected from all *S*. *pneumoniae*-infected subjects (n = 23), all non-*S*. *pneumoniae*-infected patients (n = 12), colonized control subjects (n = 2) and randomly selected healthy controls negative for *S*. *pneumoniae* colonization (n = 10). Patient demographic information and symptoms of infected subjects are presented in Table 1.

### ELISpot assays detect host anti-*S*. *pneumoniae* responses

**Fig 1:**
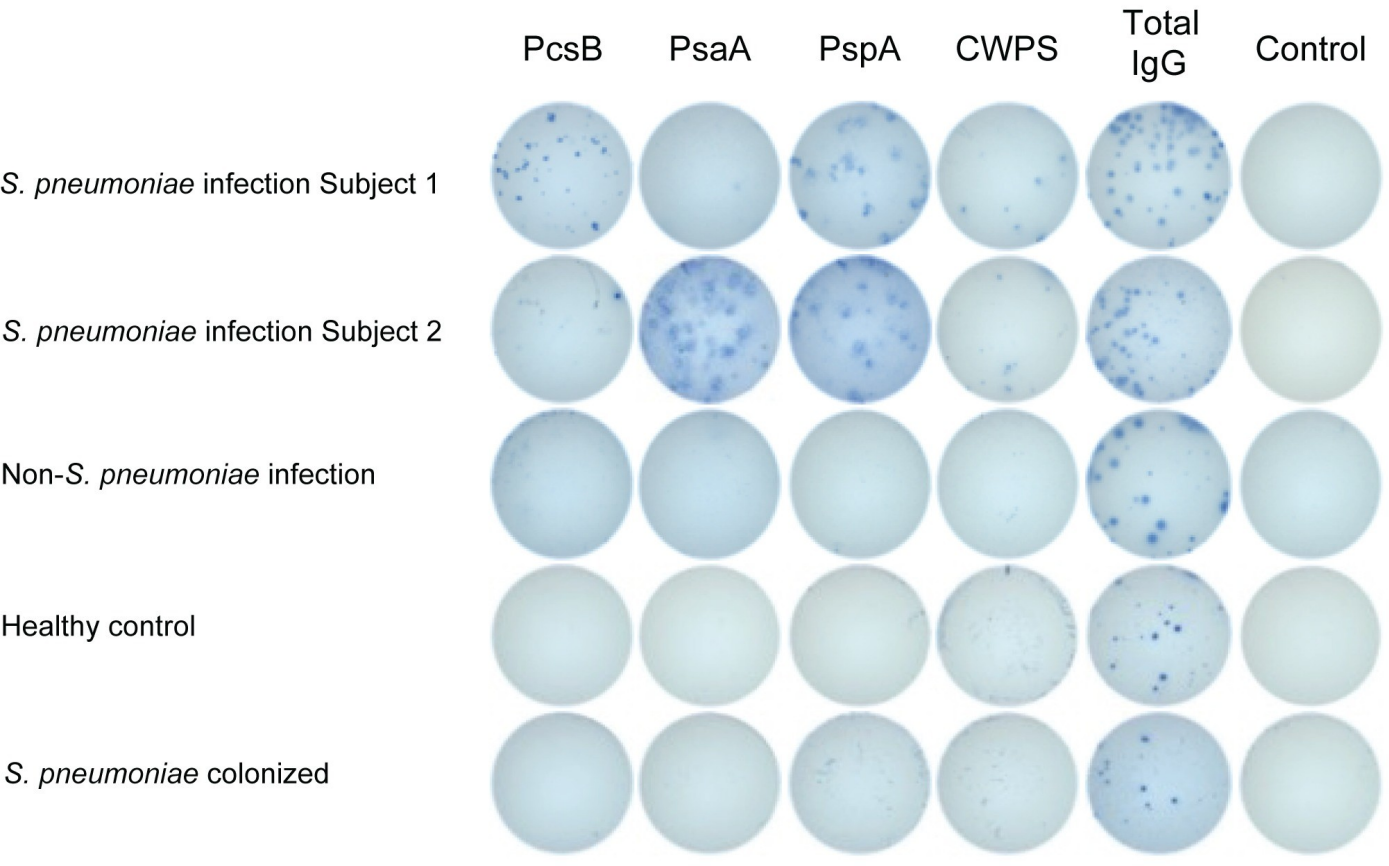
Representative ELISpot results. Peripheral blood mononuclear cells prepared from whole blood samples from five patients were tested in the ELISpot assay for antibody secreting cells producing antibodies specific for the antigens indicated at the top (PcsB, PsaA, PspA, CWPS) in the first four columns. Wells presented in the fifth column are coated with anti-IgG so they will detect any cell secreting IgG antibodies; wells in the sixth column are coated with only the blocking agent (2% BSA) so they detect only antibodies reactive with the wells in the absence of any specific antigen. The five patients are: 1,2) two with *S*. *pneumoniae* infections (top two rows; SP3 and SP10 from Fig 2A), 3) patient with infection caused by a non-*S*. *pneumoniae* pathogen (NSP5 from Fig 2A); 4) healthy control (H10 from Fig 2A); and 5) patient colonized by, but not infected with, *S*. *pneumoniae* (COL1, Fig 2A).

Representative ELISpot images from each of these 4 subject groups demonstrate evidence of IgG ASC in all groups and illustrate antigen-specific responses from circulating ASC only in the *S*. *pneumoniae*-infected patients (Fig 1).

### *S*. *pneumoniae*-specific ELISpot assays in *S*. *pneumoniae* infections and non-*S*. *pneumoniae* infection controls

**Fig 2:**
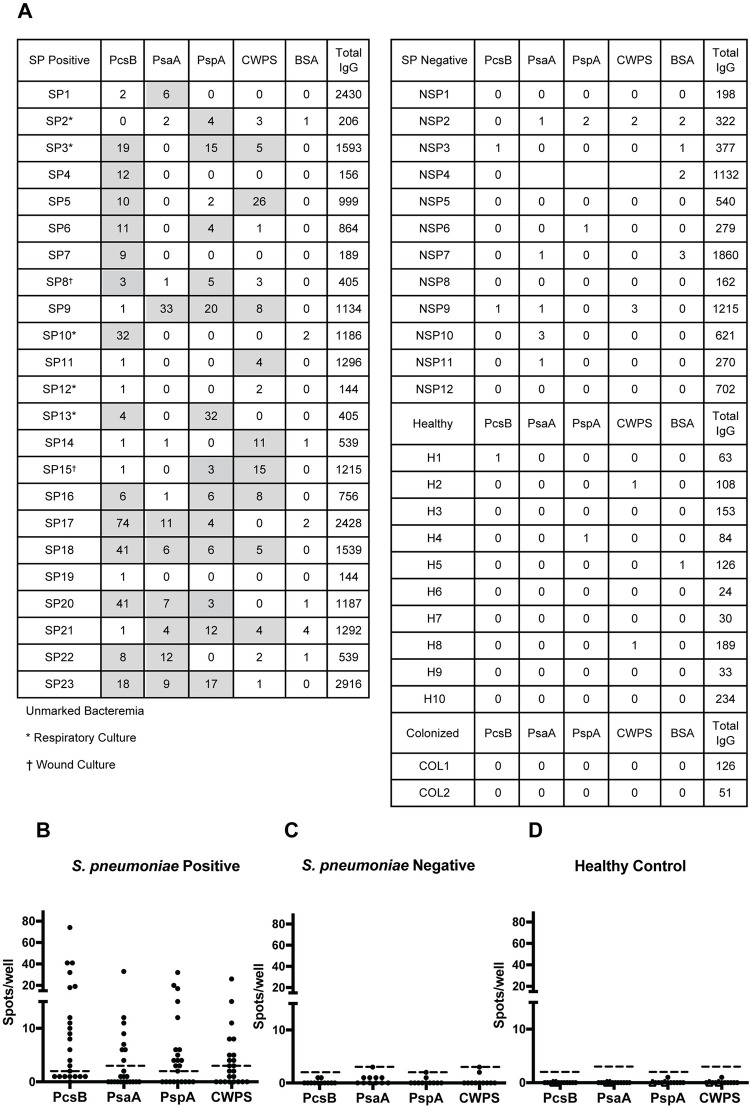
Antibody secreting cells produce antibodies specific for the four *S*. *pneumoniae* antigens in blood of patients with *S*. *pneumoniae* infections. Numbers of IgG-secreting cells (per 500,000 cells) specific to antigens (PcsB, PsaA, PspA, CWPS) in blood samples of patients with *S*. *pneumoniae*-infection (SP1-23), patients with non-*S*. *pneumoniae*-infection (NSP1-12), healthy controls with *lytA*-negative nasal swabs (H1-10), and *lytA*-positive nasal swabs (COL1-2) as measured by ELISpot (A). Spot numbers greater than C_0_ for each antigen are highlighted in gray. Summary of the number of spots in ELISpot immunoassays positive for each of four antigens in patients with *S*. *pneumoniae* infection (B; n = 23), patients with non-*S*. *pneumoniae* infection (C; n = 12) and healthy controls (D; n = 12). Healthy controls include subjects colonized (open triangles; n = 2) and not colonized (closed circles; n = 10) as determined by *lytA* PCR. Each data point represents one patient’s response specific for the indicated antigen in the number of spots/well minus background. The dashed horizontal lines indicate the diagnostic value, C_0_, the cut-off value for positive responses. The four antigens tested are listed on the x-axis, whose C_0_ were PcsB:2, PsaA:3, PspA:2, CWPS:3.

IgG ELISpot assays were used to compare the detection of ASC specific for *S*. *pneumoniae* antigens in all four groups (Fig 2A). Among patients with *S*. *pneumoniae* infections, mean spot totals for each antigen were highest for PcsB (12.9 spots/well, 95% CI 5.5 to 20.4) followed by PspA (5.8 spots/well, 95% CI 2.4 to 9.1), CWPS (4.3 spots/well, 95% CI 1.7 to 6.8), and PsaA (4.0 spots/well, 95% CI 1.0 to 7.1) (Fig 2B). Among patients in the non-*S*. *pneumoniae*-infected population, none of the patients had more than four spots/well in the four *S*. *pneumoniae* antigens tested (Fig 2C). We used the Poisson distribution to model the counts from the non-*S*. *pneumoniae* group and calculated the C_0_ value so that the Poisson test p-value is less than 0.01. For IgG, the C_0_ for antigens PcsB, PsaA, PspA, CWPS were 2, 3, 2, 3, respectively. The C_0_ distinguished subjects with documented *S*. *pneumoniae* infections from healthy controls, colonized subjects and those infected with other pathogens.

Overall, 91% (21/23) of patients with *S*. *pneumoniae* infections tested positive for at least one of the four antigens tested (Table 2). PcsB had the highest rate of detection (61%, 14/23) followed by PspA (57%, 13/23), CWPS (39%, 9/23), and PsaA (35%, 8/23). Among patients with non-*S*. *pneumoniae* infections, none (0/12) were positive for any of the antigens tested.

**Table 2:** Diagnostic performance of antibody isotypes against *S*. *pneumoniae* cell wall antigens.

	*S*. *pneumoniae* infection		Non-*S*. *pneumoniae* infection	
Antibody Isotype	Positive Test	Negative Test	Positive Test	Negative Test
IgG	21/23 (91%)	2/23 (9%)	0/12 (0%)	12/12 (100%)
IgA	15/20 (75%)	5/20 (25%)	2/9 (22%)	7/9 (78%)
IgM	5/22 (23%)	17/22 (77%)	1/11 (9%)	10/11 (91%)

Positive test defined as spot number greater than C_0_ in any single well of the four antigens tested.

### *S*. *pneumoniae*-specific IgG ELISpot assays in healthy controls and colonized subjects

To determine the background response in healthy individuals, we tested ASC from 10 healthy, non-colonized subjects and the two colonized subjects. For both healthy subject groups, no more than 1 spot/well was detected for any of the antigens tested (Fig 2D). All healthy controls and colonized subjects were negative.

### IgG ELISpot assays are more sensitive and specific than those for IgM or IgA

All PBMC specimens from patients with bacteremia were screened for IgM, IgG, and IgA antibody responses specific for the four *S*. *pneumoniae* cell wall antigens. Among the antibody isotypes, the sensitivity of the IgG assay was greater (91%, 21/23) than either IgA (75%, 15/20) or IgM (23%, 5/22) in detecting *S*. *pneumoniae* infections (Table 2). IgG (100%) had a superior specificity compared to IgA (78%). In addition, all patients positive for *S*. *pneumoniae*-specific IgA or IgM were also positive for *S*. *pneumoniae*-specific IgG. Thus, in the following experiments, we based the diagnostic assay on the detection of antigen-specific IgG alone.

### *S*. *pneumoniae*-specific IgG response is highest during the first four days following confirmation

**Fig 3:**
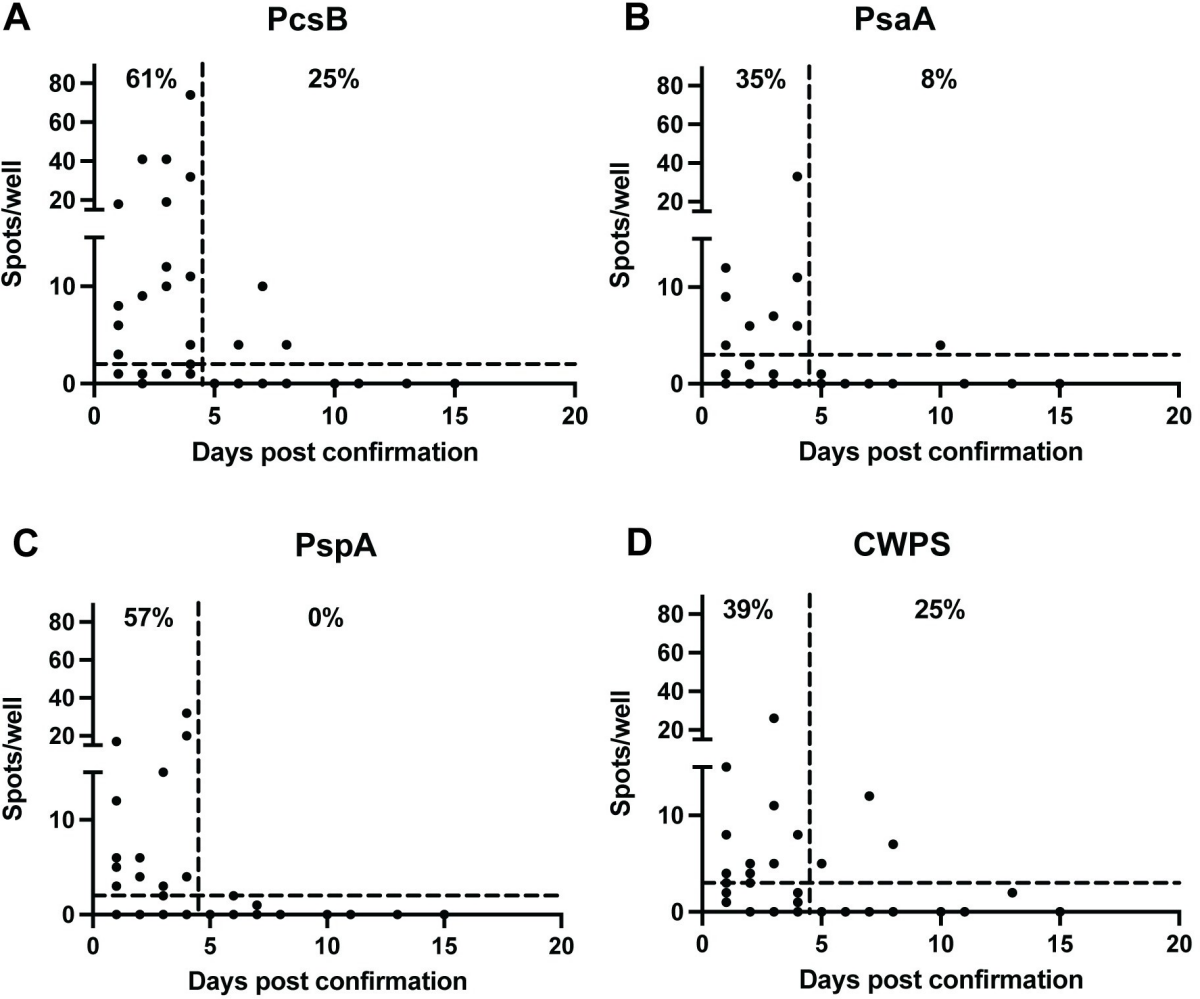
Circulating antibody secreting cells are detectable primarily in the first four days post-confirmation. Time course of pathogen-specific IgG responses to *S*. *pneumoniae* antigens PcsB (A), PsaA (B), PspA (C), and CWPS (D). Days of sample collection are presented on the x-axis as days post-confirmation from the Emory Hospital Medical Microbiology Laboratory. The dashed horizontal line represents the C_0_, the minimum number of spots for a positive sample. The C_0_ was determined for each antigen as PcsB:2, PsaA:3, PspA:2, CWPS:3. The dashed vertical line indicates Day 4.5, the observed time after which most samples no longer had circulating ASC.

To identify the optimal time for diagnostic sample collection, blood samples were collected 1-17 days following a positive blood culture. Analysis of the time course of the pathogen-specific IgG response showed that most of the ASC response occurred during the first four days following culture confirmation and declined thereafter (Fig 3A–3D). Between 35 and 61% of patients produced ASC secreting IgG for each antigen during the first four days following confirmation; however, after day 5, the frequency of positive ASC decreased substantially (0-25%). In *S*. *pneumoniae* infection, it is often difficult to know the timing of inoculation; however, we identified symptom onset based on the history. On average, patients presented four days after symptom onset. Blood cultures were sent at presentation and cultures were confirmed positive approximately 48 hours later. Patients were recruited within four days of confirmation, which was on average 10 days from time of symptom onset, with a range of 5-19 days.

### Using all four antigens detects more positive patients

**Fig 4:**
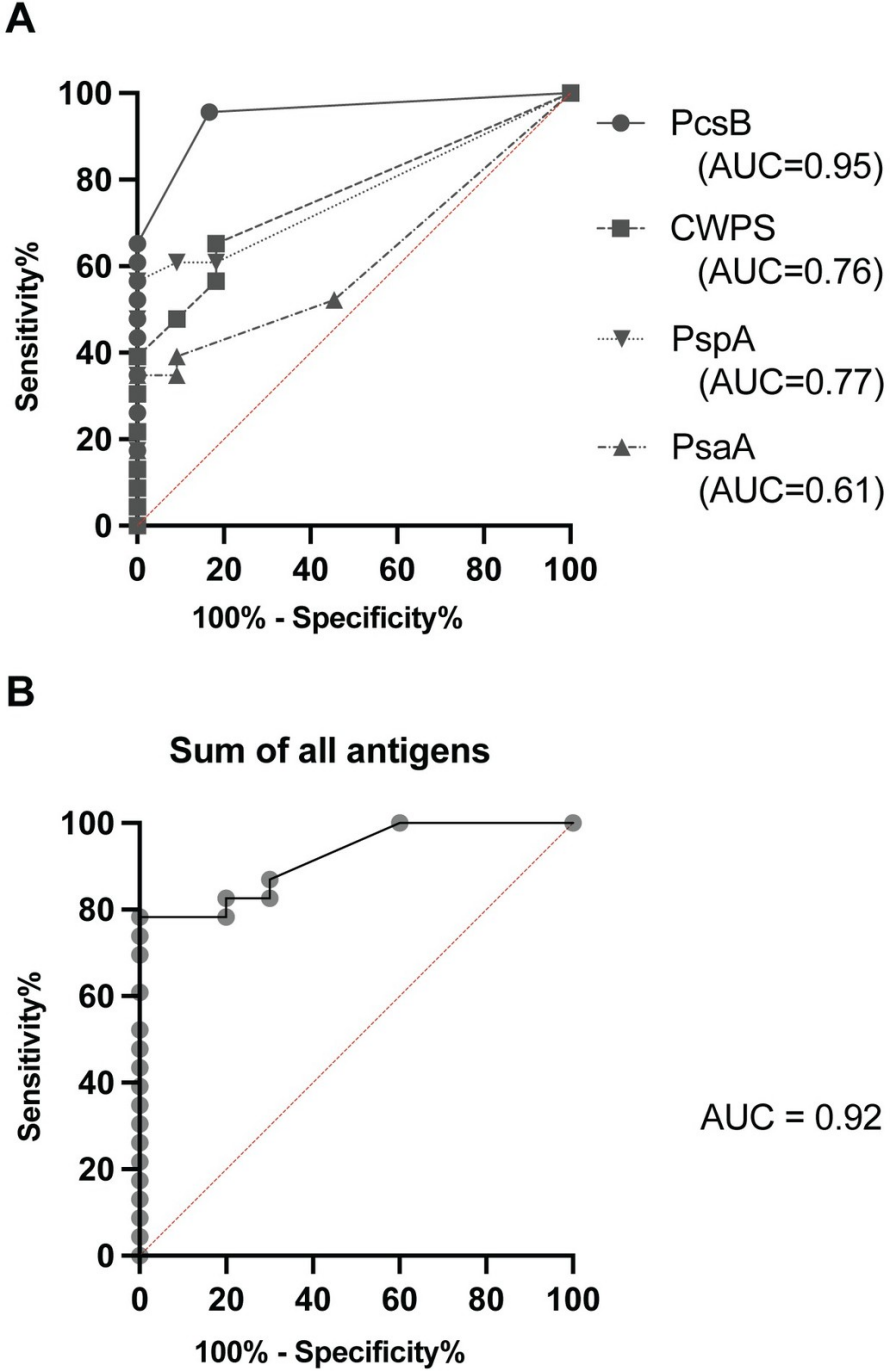
Receiver Operating Characteristic (ROC) curves for the discernment of patients with *S*. *pneumoniae* infections from patients with non-*S*. *pneumoniae* infections. ROC curves for the individual antigens PcsB (AUC = 0.95; circle), PsaA (AUC = 0.61; upright triangle), PspA (AUC = 0.77; inverted triangle), CWPS (AUC = 0.76; square)(A), and an ROC curve based on sum of the spots minus background for all antigens (B; AUC = 0.92). The diagonal red lines indicate no diagnostic value (AUC = 0.5); a perfect diagnostic test would have an AUC of 1.0.

Receiver operating characteristic (ROC) curves for the four antigens show that PcsB had the highest area under the curve, AUC = 0.95, while PsaA had the lowest (AUC = 0.61; Fig 4A). After observing that *S*. *pneumoniae*-infected patients presented with different patterns of antibody responses to the four antigens (Fig 2A), we hypothesized that the diagnostic test accuracy could be improved by summing the number of spots from all four antigen wells within a single sample. Conservatively, the non-zero y-intercept, which indicates the spot number with the highest sensitivity without any false positives, increased to 80% compared to 62% for PcsB alone, showing the benefit of having multiple antigens for the detection assay (Fig 4B). While anti-PcsB IgG alone was a strong predictor of *S*. *pneumoniae* infection (14/23), the addition of the three other antigens enabled the identification of seven additional positive patients (21/23).

### IgG ELISpot data correlates with antigen-specific antibody levels in MENSA

**Fig 5:**
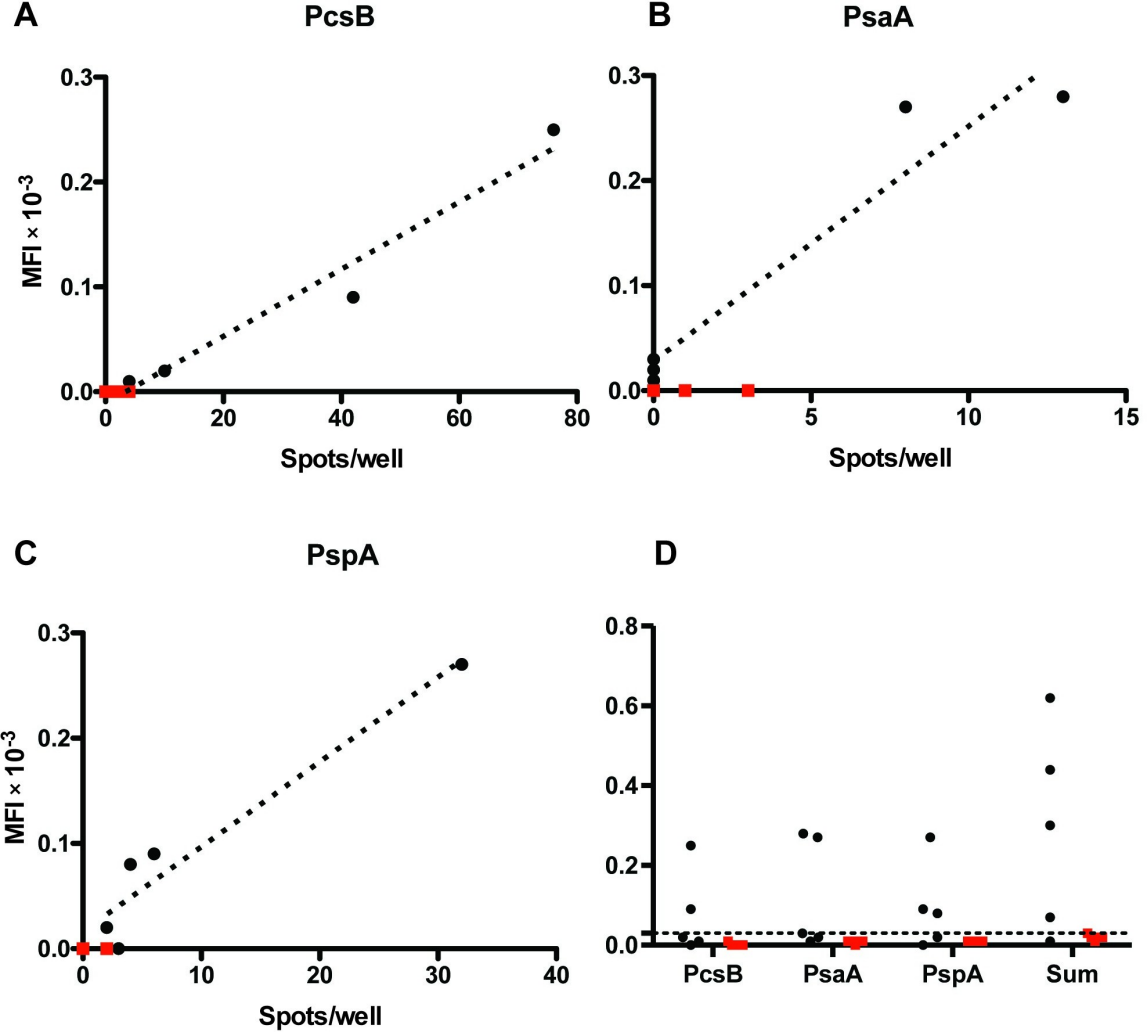
Responses in Medium Enriched with Newly Synthesized Antibodies (MENSA) tests correlate with spots/well in ELISpot immunoassays. Correlation of anti-PcsB (A), anti-PsaA (B), and anti-PspA IgG (C) responses in MENSA by Luminex immunoassay measurements (median fluorescence intensity, MFI×10^-3^) and ELISpot counts (spots/well). Black dots represent measured values for the five *S*. *pneumoniae*-positive patients; red squares represent the measured values for samples from five *S*. *pneumoniae*-negative patients. Summary of MENSA responses measured in each patient for each antigen and for the sum of all antigens (D). The highest value observed for any antigen or their sum among the *S*. *pneumoniae-*negative patients is represented as the C_0_ for MENSA samples (dashed horizontal line).

Finally, we evaluated a multiplex Luminex assay to detect *S*. *pneumoniae*-specific antibodies in MENSA, a novel matrix, as an alternative to ELISpot assays. To increase the specificity of the assay, preformed antibodies present in serum were excluded by extensive washing and ASC were placed in fresh media so only newly synthesized antibodies would be present. MENSA from patients with *S*. *pneumoniae* infections (n = 5) and patients with non-*S*. *pneumoniae* infections (n = 5) were tested for IgG-specific for the three antigens PcsB, PsaA and PspA. Antigen-specific antibody levels correlated well with IgG ELISpot assays for each individual antigen with R^2^ values of 0.92 for PspA (*p* = 0.009, Fig 5C), 0.92 for PsaA (*p* = 0.089; Fig 5B), and 0.96 for PcsB (*p* = 0.003; Fig 5A). In fact, 4 of 5 patients produced MENSA IgG antibodies reactive with one of the three antigens tested (Fig 5D) and the single patient who was not detected was positive only for the CWPS antigen that was not included in this MENSA test (Fig 2A, patient SP15). In addition, antigen-specific antibody levels were consistently negative in the MENSA collected from five patients with non-*S*. *pneumoniae* bacteremias (Fig 5D).

## Discussion

In this proof-of-concept study, we have examined the utility of circulating ASC as biomarkers for ongoing *S*. *pneumoniae* infections in human patients. The initial objective was identification of patients with acute infection, and as we predicted, *S*. *pneumoniae*-infected patients had higher frequencies of antigen-specific ASC in the blood than healthy controls, colonized subjects or patients infected with non-*S*. *pneumoniae* pathogens. Using the ASC response against only four antigens, we were able to distinguish *S*. *pneumoniae*-infected patients from healthy controls (AUC = 0.98) and from non-*S*. *pneumoniae*-infected patients (AUC = 0.92).

### The selection of antigen targets for these immunoassays

*S*. *pneumoniae* has over 90 serotypes that can be distinguished by antibody responses to carbohydrate antigens [28]. However, several proteins from *S*. *pneumoniae* are highly conserved among the 90 serotypes [29]. We selected four candidate antigens based on demonstrated immunodominance and the ability to distinguish between infection and colonization [30]. These proteins included the pneumococcal surface adhesin A (PsaA), the protein required for cell wall separation of group B streptococci (PcsB), and pneumococcal surface protein A (PspA). PsaA is a lipoprotein that functions as a host cell adhesin [31]. PcsB is an essential hydrolase that mediates the separation of dividing cells [32]. PspA is a surface protein that inhibits complement activation thereby contributing to virulence [33]. Of these, anti-PspA and anti-PcsB are most frequently detected in serum responses [34–36]. In addition, we examined the response to the *S*. *pneumoniae* cell wall polysaccharide (CWPS) which is a highly conserved teichoic acid-like polysaccharide present in the cell wall and elicits a non-protective antibody response [37]. Because it is common across multiple serotypes, it was ideal for this assay.

All four antigens had significant diagnostic utility with the highest for PcsB (AUC = 0.95) and lowest for PsaA (AUC = 0.61). The combination of all four pneumococcal antigens significantly improved the sensitivity of the assay when using a conservative C_0_ value where specificity was 100%. This approach of seeking active ASCs may also differentiate the diagnosis of *S*. *pneumoniae* from taxonomically similar commensal species, such as *S*. *mitis* and *S*. *pseudopneumoniae* [38].

There was variability of the number of ASC for each *S*. *pneumoniae* antigens, highlighting the difficulty and importance of antigen selection. This could be due to differences among the patient cohort including: (1) phase of infection (during the growth phase of *S*. *pneumoniae*, the host may encounter increased PcsB), (2) sites of infection, and (3) variations among strains (e.g., sequence variations in target antigens and expression of alternative but functionally related proteins). There is sufficient variability among hosts and pathogens that selection of antibody response to single antigen may not always be sufficient. Thus, an ASC diagnostic assay utilizing a selection of antigens serves in a practical way to enhance analytic sensitivity. Hence the capability of detecting multiple antigens from the same ASC matrix in the MENSA platform makes it an ideal system for this diagnostic test.

### Kinetics of ASC with bacterial infection

The levels of circulating ASC do not remain elevated indefinitely. Rather, ASC emerge into the bloodstream early in the course of the infection and decline to zero when the infection is resolved. This feature was evident in Fig 3 where ASC levels declined to background in most patients, and for most antigens, five days post-confirmation, plausibly reflecting successful antibiotic therapy initiated 4-5 days earlier. The decline in circulating ASC upon the resolution of infection further validates this approach for diagnosis of acute primary infection as well as potentially providing a biomarker of therapeutic efficacy. Since the assay is agnostic to pre-existing serum levels, it can be used for repeat infections in settings especially when serum titers are elevated [16, 20].

### Limitations and future objectives

A limitation of the study was the modest population size with only 23 *S*. *pneumoniae-*positive patients and 12 *S*. *pneumoniae*-negative patients. Additionally, after surveying 91 subjects, we were only able to find two *S*. *pneumoniae*-colonized subjects. This may be in part due to the prevalence of pneumococcal vaccines contributing to a lower frequency of colonized subjects. A study in the United Kingdom found only 2.8% pneumococcal carriage in parents of young children during 2015/2016 after the introduction of PCV13 in 2010 [39]. Thus, larger study numbers will enable greater scrutiny of the specificity of each antigen as a potential diagnostic target and expanding the study to include children with invasive pneumococcal disease and children heavily colonized with *S*. *pneumoniae* may also be important.

### Earlier sample timing may yield improved sensitivity

In the present study, we only analyzed samples collected 1-4 days post-confirmation or 5-9 days post-clinical presentation for most subjects. Based on the data presented in Fig 3, ASC collected after day 4 are likely to be of limited value for primary diagnosis, however, samples from earlier time points, closer to symptom onset and clinical presentation, may have higher sensitivity.

### Advantages of MENSA-based assays

ELISpot or MENSA-based diagnostic methods currently take at least 24 hours; thus, improving cell preparation and dramatically reducing incubation time for ELISpot assays or antibody accumulation in MENSA are plausible improvements for making this method practical for clinical labs. Compared with the ELISpot assay, the MENSA/Luminex assay requires significantly fewer cells since the assay can detect antibodies to multiple antigens at the same time. Additionally, the MENSA assays are readily amenable for higher throughput and storage for later studies. Thus, MENSA from unidentified pneumonias can be stored and tested for emerging pathogens identified in the future.

### Utilization of ASC as a diagnostic test for viral and bacterial pathogens

In this study, we show that by assessing circulating ASC, diagnosis of *S*. *pneumoniae* bacteremic infections is possible. Similarly, circulating ASC as a diagnostic markers have been demonstrated for viral and bacterial pathogens including: Influenza virus [16, 26, 40, 41], Respiratory syncytial virus [17], *Salmonella enterica* [42], *Mycobacterium tuberculosis* [43–45], *Staphylococcus aureus* [18, 20], and *Clostridioides difficile* [19]. Based on the other opportunities for measuring ASC in the circulation, we anticipate that other indications of *S*. *pneumoniae* (e.g., pneumonia, meningitis) and other pathogens that cause bacteremia (e.g., Staphylococci, Enterococci) will be addressable in a similar way.

## Conclusion

We present a simple method for diagnosing adult patients with *S*. *pneumoniae* infection. The method measures circulating ASC that emerge into blood early in an infection and rapidly disappear from circulation following its resolution. If proven to be more accurate and practical than routine diagnostic methods currently used, this novel immunoassay, with modest improvements, could substantially advance bacterial diagnostics.

## Abbreviations

ASC: Antibody-secreting cells
CAP: Community acquired pneumonia
CWPS: *S*. *pneumoniae* cell wall polysaccharide
ELISpot: Enzyme-linked immunospot
MENSA: Medium enriched for newly synthesized antibodies
PcsB: Protein required for cell wall separation of group B streptococcus
PsaA: Pneumococcal surface adhesin A
PspA: Pneumococcal surface protein A
*S*. *pneumoniae*: *Streptococcus pneumoniae*
